# Effects of behavioural activation on the neural basis of other perspective self-referential processing in subthreshold depression: a functional magnetic resonance imaging study

**DOI:** 10.1017/S0033291716002956

**Published:** 2016-11-29

**Authors:** S. Shiota, Y. Okamoto, G. Okada, K. Takagaki, M. Takamura, A. Mori, S. Yokoyama, Y. Nishiyama, R. Jinnin, R. I. Hashimoto, S. Yamawaki

**Affiliations:** 1 Department of Psychiatry and Neurosciences, Graduate School of Biomedical and Health Sciences, Hiroshima University, Hiroshima, Japan; 2 Department of Language Sciences, Graduate School of Humanities, Tokyo Metropolitan University, Tokyo, Japan; 3 Medical Institute of Developmental Disabilities Research, Showa University, Tokyo, Japan

**Keywords:** Behavioural activation, dorsal medial prefrontal cortex, monitoring function, subthreshold depression

## Abstract

**Background:**

It has been demonstrated that negatively distorted self-referential processing, in which individuals evaluate one's own self, is a pathogenic mechanism in subthreshold depression that has a considerable impact on the quality of life and carries an elevated risk of developing major depression. Behavioural activation (BA) is an effective intervention for depression, including subthreshold depression. However, brain mechanisms underlying BA are not fully understood. We sought to examine the effect of BA on neural activation during other perspective self-referential processing in subthreshold depression.

**Method:**

A total of 56 subjects underwent functional magnetic resonance imaging scans during a self-referential task with two viewpoints (self/other) and two emotional valences (positive/negative) on two occasions. Between scans, while the intervention group (*n* = 27) received BA therapy, the control group (*n* = 29) did not.

**Results:**

The intervention group showed improvement in depressive symptoms, increased activation in the dorsal medial prefrontal cortex (dmPFC), and increased reaction times during other perspective self-referential processing for positive words after the intervention. Also, there was a positive correlation between increased activation in the dmPFC and improvement of depressive symptoms. Additionally, there was a positive correlation between improvement of depressive symptoms and increased reaction times.

**Conclusions:**

BA increased dmPFC activation during other perspective self-referential processing with improvement of depressive symptoms and increased reaction times which were associated with improvement of self-monitoring function. Our results suggest that BA improved depressive symptoms and objective monitoring function for subthreshold depression.

## Introduction

Subthreshold depression is defined as clinically significant depressive symptoms which do not fulfill diagnostic criteria for a major depressive episode (Bertha & Balazs, 2013). Subthreshold depression can lead to serious functional impairment, including a negative impact on academic performance and social activity, particularly in adolescence (Bertha & Balazs, 2013). Subthreshold depression is associated with an elevated risk of developing a major depressive episode (Bertha & Balazs, 2013).

Given these concerns, it is very important to elucidate the pathogenic mechanisms underlying subthreshold depression and to develop effective interventions. Although pharmacotherapy is widely provided, use of selective serotonin reuptake inhibitors in youth can have aversive effects, such as increased suicidality (Hammad *et al.* 2006). Consequently, psychotherapy is preferred for treatment of youth depression. Cognitive–behavioural therapy (CBT) has demonstrated efficacy with this population (Cuijpers *et al.* 2007).

In particular, behavioural activation (BA) is one key component of CBT for depression, and is an effective intervention for the treatment of major depression (Jacobson *et al.* 1996). Based on the approach of Lewinsohn & Grosscup (1980), BA is focused on enhancement of self-monitoring, increasing healthy goal-oriented behaviour, and increasing environmental reward frequency. In the course of BA interventions, participants monitor and assess their daily activities and work to change their habitual behaviours, such that pleasant events are increased and depressive symptoms are improved (Jacobson *et al.* 1996). Recently, we carried out a randomized controlled trial of BA for subthreshold depression in college students, demonstrating that BA is an effective intervention for people with subthreshold depression (Takagaki *et al.* 2016). However, brain mechanisms underlying BA intervention are not fully understood.

A few studies have investigated BA-related brain mechanisms in depression using tasks that probe emotion regulation (Dichter *et al.* 2010) and reward processing (Dichter *et al.* 2009). Our previous study demonstrated that BA for subthreshold depression leads to functional changes in the left ventrolateral prefrontal cortex (PFC) and angular gyrus during loss anticipation in a monetary incentive task (Mori *et al.* 2016).

Material related to the self is more readily remembered than material related to others, which is referred to as self-referential processing (Rogers *et al.* 1977; Symons *et al.* 1997). Especially, self-referential processing with other perspective is related to a person's objective monitoring function (Davis *et al*. 1996; Galinsky & Ku, 2004). It has been demonstrated that negatively distorted self-perspective self-reference, in which individuals evaluate one's own self using self-viewpoint, is a pathogenic mechanism in both major depression and subthreshold depression (Kuiper & Derry, 1982), and it has been suggested that there are abnormalities in brain activation that accompany such negatively distorted self-perspective (Calni *et al.* 2004; Yoshimura *et al.* 2010). In addition, individuals with depression also show impairments in positive other perspective self-reference, where individuals evaluate one's own self using his or her friend's viewpoint (Surguladze *et al.* 2004), and such altered self-referential processing leads to the maintenance of depressive symptoms (Weightman *et al.* 2014). Our previous study demonstrated that symptom improvement in CBT was associated with activation changes in the medial PFC (mPFC) during negative self-reference from the self-perspective in depression (Yoshimura *et al.* 2014).

The original purpose of BA was to increase access to positively reinforcing activities, increasing the availability of rewards and modifying behaviours to elicit changes in depressive symptoms. However, according to a previous study (Jacobson *et al.* 1996), BA also significantly improved the score in the Automatic Thoughts Questionnaire (ATQ) related to the self-concept. The ATQ assesses a person's negative automatic thoughts related to negative self-concepts (Hollon & Kendall, 1980). Based on these findings, we speculated that BA might modify the self-concept. A notable point of the current study was that participants repeatedly evaluated their behaviours and their results during the behavioural experiments. Lewinsohn & Grosscup (1980) have suggested that participants enhance their monitoring function as a consequence of repeated evaluation of their own behaviours and results during BA. Self-referential processing with an other perspective is related to a person's objective monitoring function (Davis *et al*. 1996; Galinsky & Ku, 2004). Therefore, we hypothesize that BA enhances other perspective self-referential processing, which is a part of the self-concept that is associated with changes in mPFC activation. However, there are no published studies that have specifically focused on brain function changes related to other perspective self-reference, which is also known to be associated with mPFC activation (Ruby & Decety, 2001) in depression and subthreshold depression during the course of treatment. In the present study, we carried out a BA intervention for university students with subthreshold depression in order to examine whether BA might modify mPFC activity during referential processing. We employed the modified self-reference functional magnetic resonance imaging (fMRI) task adopted by Hashimoto *et al.* (2016) which was designed to examine effects of perspective on brain activation for self- and other referential processing. We hypothesized that successful BA intervention may (1) decrease negative other perspective self-reference, corresponding to mPFC activation and (2) increase positive other perspective self-reference, also corresponding to mPFC activation.

## Method

### Participants

Participants were recruited over a 2-year period between 2013 and 2014 from Hiroshima University, by screening with the Japanese version of the Beck Depression Inventory-II (BDI-II) (Kojima & Furukawa, 2003) and the Japanese version of the structured clinical interview (Composite International Diagnostic Interview; Kawakami *et al.* 2005). The inclusion criterion was BDI-II scores greater than or equal to 10. The exclusion criteria were major depressive episode within the past year, lifetime history of bipolar disorder, presently undergoing psychopharmacological or psychological treatment, and the possibility of an acute suicide attempt. A total of 61 students (mean age = 18.2 years, s.d. = 0.4 years, 40 males, 21 females) with subthreshold depression were enrolled in the randomized controlled trial of BA in the second year. Individuals were then randomly allocated to an intervention group (*n* = 30, mean age = 18.2 years, s.d. = 0.4 years, 19 males, 11 females) or to a no-treatment control group (*n* = 31, mean age = 18.2 years, s.d. = 0.4 years, 21 males, 10 females). One participant in the intervention group and another in the no-treatment control group did not participate in the fMRI study. In all, 29 participants in the intervention group (mean age = 18.2 years, s.d. = 0.4 years, 19 males, 10 females) and 30 participants in the no-treatment control group (mean age = 18.2 years, s.d. = 0.4 years, 21 males, nine females) participated in the fMRI study. Three participants were excluded from fMRI analyses, because two of them took medication and the other did not yield usable data. All told, fMRI and behavioural data of 27 participants in the intervention group and 29 individuals in the control group were analysed. The ethics committee of Hiroshima University approved the study protocol. Prior to the study, written informed consent was obtained from all participants.

### Experimental design

Freshmen attending Hiroshima University were recruited for the randomized controlled trial (Takagaki *et al.* 2016). A total of 59 subjects underwent fMRI scans during a referential task with two viewpoints (self/other) and two emotional valences (positive/negative) (Hashimoto *et al.* 2016). For the intervention group, the scan occurred both before and after BA intervention, and the control group was also scanned twice, with an interval of 5 weeks without any intervention.

### Treatment protocol

Individuals in the intervention group participated in the BA programme (Takagaki *et al.* 2016). The BA intervention is described in detail elsewhere (Takagaki *et al.* 2016). Briefly, the BA intervention included five weekly sessions, each of 60 min duration. In the 1st session, participants received psycho-education about depression and BA and set long-term and short-term goals. The concept of activity monitoring was introduced. In the 2nd session, participants developed a hierarchy of about 10 tasks, were introduced to the concept of behavioural experiments, and received instruction around how to increase scheduled activities. The 3rd and 4th sessions continued to focus on behavioural experiments and increasing scheduled activities. In the 5th session, participants assessed improvements in activity-monitoring ability compared with the 1st session, reviewed progress to date, and built a plan for self-management in stressful situations. This treatment protocol involved homework for all sessions. Participants monitored their behaviours during all 5 weeks of the programme.

### Evaluation

Effects of BA on other perspective self-referential processing were measured using an fMRI task. Behavioural data included reaction times and judgment ratios. Reaction time was the average duration of participants' responses to stimuli and judgment ratio was the average number of ‘yes’ response counts during each emotional valence (positive/negative) condition, as described below. Depression was assessed using the BDI-II at pre- and post-treatment. Additionally, we calculated Δbrain activation (post-treatment minus pre-treatment) and percentage change on BDI-II [(pre-treatment minus post-treatment) divided by pre-treatment], as well as percentage change on reaction times [(post-treatment minus pre-treatment) divided by pre-treatment] and percentage change on judgment ratios [(post-treatment minus pre-treatment) divided by pre-treatment]. We used percentage change scores in the present study because of their merits for correlation analyses. Reaction times, brain activation and BDI-II scores had different value ranges and the percentage change score can adjust for differences in the range of value of different variables (Tan & Michel, 2011). Providing information related to the content and precision of scores helps the meaningful interpretation of raw scores (Tong & Kolen, 2010). Analysis of data from clinical trials often uses percentage change scores and raw change scores to adjust treatment responses to the baseline (Kaiser, 1989). Additionally, the percentage change score can be adapted for ratio scales (Russell, 2000). If the variable were a ratio scale, then pre-scores and changed scores would be correlated (Sugimoto, 2008). We performed correlation analyses that included pre-scores and changed scores for different variables. Furthermore, we assessed normal distribution using skewness and kurtosis (Kim, 2013). It is known that distribution of small samples (*n* < 50) would be non-normal if the absolute *z*-scores for either skewness or kurtosis were larger than 1.96 (Kim, 2013). To examine intervention effects, we performed correlation analyses that included percentage change on BDI-II and Δbrain activation, percentage change on reaction times, and percentage change on judgment ratios, respectively.

### fMRI task

The fMRI task (Hashimoto *et al.* 2016) included five judgment conditions: other perspective self-judgment condition (OS); other perspective other judgment condition (OO); self-perspective self-judgment condition (SS); self-perspective other judgment condition (SO); and control judgment (word recognition) condition (Cont). Before the first scanning, participants were asked to name three of their friends of the same gender. Next, participants ranked these friends according to closeness. The second closest friend was used as the ‘other’ during the experiment. In OS condition trials, participants judged the presented positive (OS-P) or negative (OS-N) trait words using his or her friend's viewpoint, as applicable to one's own self [e.g. ‘Does Bob think you are calm?’ (OS-P)]. In OO condition trials, participants judged the presented positive (OO-P) or negative (OO-N) trait words using his or her friend's viewpoint, as applicable to his or her friend [e.g. ‘Does Bob think Bob is calm?’ (OO-P)]. In SS condition trials, participants judged positive (SS-P) or negative (SS-N) trait words using the self-viewpoint, applicable to one's own self [e.g. ‘Do you think you are calm?’ (SS-P)]. In SO condition trials, participants judged positive (SO-P) or negative (SO-N) trait words using the self-viewpoint, as applicable to his or her friend [e.g. ‘Do you think Bob is calm?’ (SO-P)]. Finally, in control (Cont) condition trials, participants judged positive (Cont-P) or negative (Cont-N) words as to whether or not they could understand their meanings. For all conditions, participants made a ‘yes’ or ‘no’ response by pressing a button with their right index or middle fingers respectively. Button presses were recorded using an MRI-compatible keypad (4 Side Button Cylinder; Current Designs, USA). The average number of ‘yes’ responses in each condition was defined as the judgment ratio (Yoshimura *et al.* 2014) and reaction time was defined as the average latency of participant's responses after adjective words were displayed on the screen (Sheppard & Teasdale, 2000). Participants performed each condition eight times, and each condition included four blocks. At the onset of each block, a fixation cross was displayed for 1000 ms, followed by an instruction cue presented for 3000 ms (e.g. ‘self-viewpoint self-judgment’). Each block consisted of five trials, each consisting of a fixation cross displayed for 1000 ms followed by an adjective displayed for 3000 ms and the participant's response. A fixation point as an intermission between blocks was displayed for 4000 ms, and then the instruction cue for the next block was presented. The duration of each block was 28 s. To control for order effects, blocks within a run were presented in a pseudo-random order, with no two consecutive blocks featuring the same instructions. The total time for the task was 1120 s. Both judgment ratio and reaction time were recorded using Presentation software (Neurobehavioral Systems, USA).

### Stimuli

Word stimuli were selected from Anderson's list of personality-trait words (Anderson, 1968) and Bochner and Van-zyl's list of personality-trait words (Bochner & Van-zyl, 1985). The words in both lists were translated into Japanese (Yoshimura *et al.* 2010, 2014; Hashimoto *et al.* 2016). The top 200 words were selected for frequency using rank of Google searches as a criterion. Each adjective word was presented once randomly during the fMRI task.

### MRI acquisition

MRI scanning was performed using a Verio 3.0T device (Siemens AG, Germany). A time-course series of 536 scans were acquired with T2*-weighted, gradient echo, echo-planar imaging sequences. Each volume consisted of 38 slices, with a slice thickness of 3.8 mm with no gap. The repetition time (TR) was 2000 ms, the echo time (TE) was 25 ms and flip angle was 80°. The field of view (FOV) was 240 mm, and the voxel size was 3.8 × 3.8 × 3.8 mm. After functional scanning, structural scans were acquired using a T1-weight gradient echo pulse sequence (TR = 2300 ms, TE = 2.98 ms, flip angle = 9°, FOV = 256 mm, voxel size = 1 ×1 × 1 mm), which facilitated localization.

### Behavioural data analysis

We conducted a three-way mixed ANOVA using group (intervention group *v.* control group) as a between-subjects factor, and time (pre-treatment *v.* post-treatment) and emotional valence (positive *v.* negative) as within-subjects factors, for judgment ratio and reaction time in the OS condition.

### fMRI data analysis

Image processing and statistical analysis were carried out using Statistical Parametric Mapping (SPM8) software (Wellcome Department of Cognitive Neurology, UK). The first five volumes of the fMRI run were discarded to allow for T1 stabilization. All of the remaining volumes were slice timing corrected, realigned to the mean volume to correct for head motion, spatially normalized using the Montreal Neurological Institute (MNI) T1 template, and smoothed with 8-mm full-width, half-maximum Gaussian filter. A whole-brain voxel-by-voxel multiple linear regression model was employed at the individual participant level. Each condition was modelled using a box-car function convolved with a canonical haemodynamic response function. The realignment parameters were also included in the models as covariates of no interest. To evaluate brain activation related to OS, OO, SS and SO conditions for both positive and negative valence, we created eight contrasts (‘OS-P minus Cont-P’, ‘OS-N minus Cont-N’, ‘OO-P minus Cont-P’, ‘OO-N minus Cont-N’, ‘SS-P minus Cont-P’, ‘SS-N minus Cont-N’, ‘SO-P minus Cont-P’ and ‘SO-N minus Cont-N’) in the first-level analysis for each participant. These contrasts were submitted to group analysis using a random-effect model. First, one-sample *t* tests were performed for all participants to assess the overall effect of each contrast, using the contrasts from pre-treatment experimental sessions. Second, two-sample *t* tests (intervention group *v.* control group) were performed by using subtraction images of contrast in the OS condition (post-minus pre-, i.e. Δbrain activation) to assess the effect of BA on brain activity. Based on our hypothesis, we used the mPFC as the *a priori* region of interest (ROI) based on previous work (Ochsner *et al.* 2005; Yoshimura *et al.* 2010, 2014). According to our hypothesis, mPFC activation was expected to change in association with changes in other perspective self-referential processing. We conducted ROI analyses using the data of a previous functional brain imaging study of other perspective self-referential processing, in order to validate our hypothesis. The ROI was defined on the basis of a functional brain imaging study by Ruby & Decety (2001) that demonstrated the involvement of ROI in other perspective self-referential tasks (6 mm radius sphere, centre at MNI coordinates *x* = 4, *y* = 50, *z* = 40). Brain activations were reported if they exceeded *p* < 0.001 (uncorrected) at the single voxel level, and *p* < 0.05 [family-wise error (FWE) small volume corrected within the ROI] at the cluster level.

## Results

### Gender difference

There were significant differences in the number of males and females (χ^2^ = 7.45, *p* < 0.05).

### Normality test

We conducted correlation analyses that included pre- and changed scores of each variable, in order to confirm if these variables were a ratio scale. In the intervention group, there were significant correlations between pre-BDI-II scores and changes in BDI-II scores (*r* = 0.63, *p* < 0.0001), and between pre-reaction time scores and changes in reaction time scores (*r* = −0.50, *p* < 0.001). In the control group, there was a significant correlation between pre-BDI-II scores and changes in BDI-II scores (*r* = 0.48, *p* < 0.001), and a significant correlation between pre-reaction time scores and changes in reaction time scores (*r* = −0.44, *p* < 0.005). Second, we conducted a normality test using skewness and kurtosis (Kim, 2013). For the intervention group – percentage change in BDI-II: skewness = 0.89 < 1.96, kurtosis = 0.008 < 1.96; percentage change in reaction time: skewness = 0.20 < 1.96, kurtosis = 0.59 < 1.96. For the control group – percentage change in the BDI-II: skewness = 3.18 > 1.96, kurtosis = 12.51 > 1.96; percentage change in reaction time: skewness = 0.54 < 1.96, kurtosis = 0.75 < 1.96. Results indicated that only the percentage change in BDI-II of the control group was not normally distributed.

### Psychological and behavioural data

Table 1 shows psychological and behavioural data. The group × time interaction was significant for the BDI-II (*F*_1,54_ = 10.359, *p* < 0.01, *η*^2^ = 0.161). Depressive symptoms were significantly improved in the BA intervention group compared with the control group. Concerning reaction times, the three-way interaction between group, time and valence was significant only in the OS condition (*F*_1,54_ = 7.896, *p* < 0.01, *η*^2^ = 0.128). *Post-hoc* analysis revealed that there was a significant group difference at pre-session in OS-N (*p* < 0.05, *η*^2^ = 0.074), but not in OS-P. In contrast, there was a significant group difference at post-session in OS-P difference (*p* < 0.05, *η*^2^ = 0.073), but not in OS-N. Therefore, the intervention group demonstrated longer reaction times at post-treatment in OS-P. There were no significant main effects or interactions in judgment ratios between group, time or emotional valence in the OS condition.

**Table 1.** Scores of BDI-II, judgment ratio and reaction time during four judgment conditions on two emotional valences at pre-/post-BA intervention

	Intervention group (*n* = 27)		Control group (*n* = 29)	
	Pre (baseline)	Post (following BA)	Pre (baseline)	Post (following BA)
BDI-II	12.7 (5.6)	7.3 (6.3)	13.7 (5.0)	12.8 (6.8)
Judgment ratio				
Other perspective self-judgment/positive (OS-P)	8.1 (3.6)	7.8 (3.7)	8.9 (2.8)	8.5 (3.4)
Other perspective self-judgment/negative (OS-N)	4.9 (3.7)	5.1 (4.0)	6.2 (3.6)	6.5 (4.4)
Other perspective other judgment/positive (OO-P)	9.6 (3.4)	9.5 (3.5)	11.0 (3.9)	11.3 (4.0)
Other perspective other judgment/negative (OO-N)	3.3 (3.7)	3.4 (2.9)	4.6 (3.2)	4.7 (2.7)
Self-perspective self-judgment/positive (SS-P)	6.0 (3.7)	6.7 (4.0)	8.0 (3.6)	7.8 (3.7)
Self-perspective self-judgment/negative (SS-N)	10.0 (3.9)	10.3 (4.6)	9.6 (3.5)	10.5 (4.0)
Self-perspective other judgment/positive (SO-P)	12.2 (3.6)	12.7 (4.0)	13.1 (3.5)	12.3 (3.4)
Self-perspective other judgment/negative (SO-N)	2.7 (2.8)	2.3 (1.8)	3.7 (3.8)	4.1 (2.8)
Reaction time, ms				
Other perspective self-judgment/positive (OS-P)	1624.9 (354.4)	1706.4 (331.4)	1579.5 (211.6)	1546.5 (226.1)
Other perspective self-judgment/negative (OS-N)	1668.1 (330.4)	1669.9 (352.1)	1514.3 (209.4)	1614.3 (278.7)
Other perspective other judgment/positive (OO-P)	1739.0 (347.4)	1734.0 (349.4)	1594.0 (263.2)	1582.0 (238.3)
Other perspective other judgment/negative (OO-N)	1632.0 (389.3)	1560.0 (333.4)	1577.0 (269.4)	1526.0 (239.4)
Self-perspective self-judgment/positive (SS-P)	1558.3 (246.6)	1548.3 (307.6)	1512.1 (198.4)	1471.9 (260.6)
Self-perspective self-judgment/negative (SS-N)	1664.3 (325.0)	1631.7 (283.4)	1525.9 (205.8)	1568.8 (264.9)
Self-perspective other judgment/positive (SO-P)	1615.0 (352.4)	1632.0 (291.7)	1524.0 (246.5)	1590.0 (276.7)
Self-perspective other judgment/negative (SO-N)	1631.0 (340.1)	1581.0 (281.1)	1469.0 (245.8)	1529.0 (261.4)

Data are given as mean (standard deviation).

BDI-II, Beck Depression Inventory-II; BA, behavioural activation.

### fMRI data

#### Overall effect of each contrast

Table 2 shows the results of whole-brain one-sample *t* tests conducted for all subthreshold depressive participants, for each contrast. In OS-P, the left superior mPFC (Brodmann area A10), left mPFC (Brodmann area 8), left angular gyrus (Brodmann area 39), right cerebellum and left precuneus (Brodmann area 31) showed significant activation. In OS-N, the left superior mPFC (Brodmann area 8), left mPFC (Brodmann area 8), left posterior cingulum (Brodmann area 23), left angular gyrus (Brodmann area 39) and right cerebellum showed significant activation. Significant mPFC activation was shown in all conditions for both positive and negative words.

**Table 2.** Brain regions exhibiting significant activation during four judgment conditions on two emotional valences

Conditions	Area	Cluster extent	Brodmann's area	Side	*Z* value	*x*	*y*	*z*
Other perspective self-judgment/positive (OS-P)	Cerebellum	1217		Right	6.22	−26	−80	−34
	Precuneus	2182	31	Left	6.17	−6	−56	34
	Angular gyrus	1315	39	Left	5.51	−44	−60	30
	Superior medial frontal gyrus	2997	10	Left	5.41	−10	52	46
	Medial frontal gyrus	559	8	Left	4.54	−34	12	40
Other perspective self-judgment/negative (OS-N)	Posterior cingulum	2142	23	Left	6.20	−10	−50	30
	Angular gyrus	868	39	Left	5.58	−40	−58	30
	Superior medial frontal gyrus	1200	8	Left	5.39	−10	40	50
	Cerebellum	456		Right	5.32	26	−80	−34
	Medial frontal gyrus	389	8	Left	4.17	−34	12	42
Other perspective other judgment/positive (OO-P)	Cerebellum	848		Right	5.86	24	−80	−32
	Precuneus	1841	31	Left	5.59	−6	−54	32
	Angular gyrus	1128	39	Left	5.43	−48	−60	26
	Superior medial frontal gyrus	2338	8	Left	5.28	−8	52	42
	Precentral gyrus	919	8	Left	4.75	−40	12	46
Other perspective other judgment/negative (OO-N)	Angular gyrus	1083	39	Left	6.48	−42	−58	28
	Precuneus	2654	31	Left	6.21	−6	−58	26
Self-perspective self-judgment/positive (SS-P)	Calcarine	1657		Right	5.23	8	−84	4
	Precuneus	1221	31	Left	4.90	−6	−54	30
	Superior frontal gyrus	1644	9	Left	4.66	−8	56	28
	Caudate	161		Left	4.07	−10	10	16
Self-perspective self-judgment/negative (SS-N)	Angular gyrus	1083	39	Left	6.48	−42	−58	28
	Precuneus	2654	31	Left	6.21	−6	−58	26
	Superior medial frontal gyrus	912	10	Left	4.45	−10	58	16
Self-perspective other judgment/positive (SO-P)	Precuneus	2413	31	Left	6.97	−6	−58	32
	Angular gyrus	1351	39	Left	5.77	−44	−60	26
	Superior medial frontal gyrus	4667	10	Left	5.64	−8	52	44
	Medial temporal pole	1193	21	Left	5.10	−56	−8	−24
Self-perspective other judgment/negative (SO-N)	Precuneus	5589	31	Left	6.08	−6	−56	28
	Medial temporal pole	373	20	Left	5.46	−58	−10	−20
	Rectus gyrus	1773	11	Left	5.43	−4	44	−14
	Angular gyrus	483	39	Left	4.60	−46	−58	26

#### Effect of BA

**Fig. 1.**
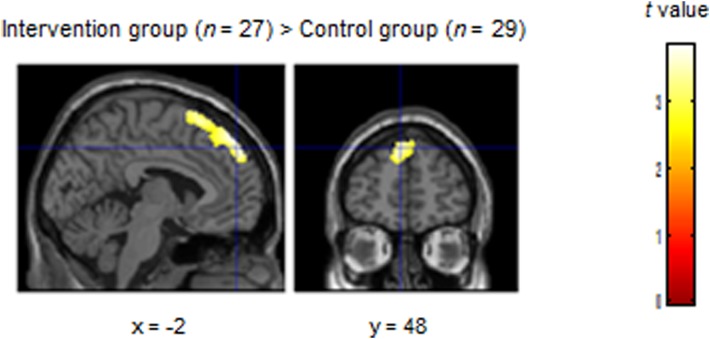
Increase in left dorsal (superior) medial prefrontal cortex (mPFC) activation during the other perspective self-judgment (OS-P) condition for positive words. The intervention group showed significantly increased activation of the left dorsal (superior) mPFC (*x* = −2, *y* = 48, *z* = 50, *t* = 3.24, cluster size = 70, cluster level family-wise error-corrected *p* = 0.016, with small volume correction) compared with the control group.

In accordance with our *a priori* hypothesis, we restricted these analyses to the mPFC. The intervention group showed a significant increase in left dorsal (superior) mPFC activation in the OS-P (*x* = −2, *y* = 48, *z* = 50, *t* = 3.24, cluster size = 70, cluster level FWE-small volume corrected *p* = 0.016) compared with the control group (see Fig. 1). There was no significant difference in OS-N activation in the mPFC between the intervention and control groups.

### Correlation analysis

**Fig. 2.**
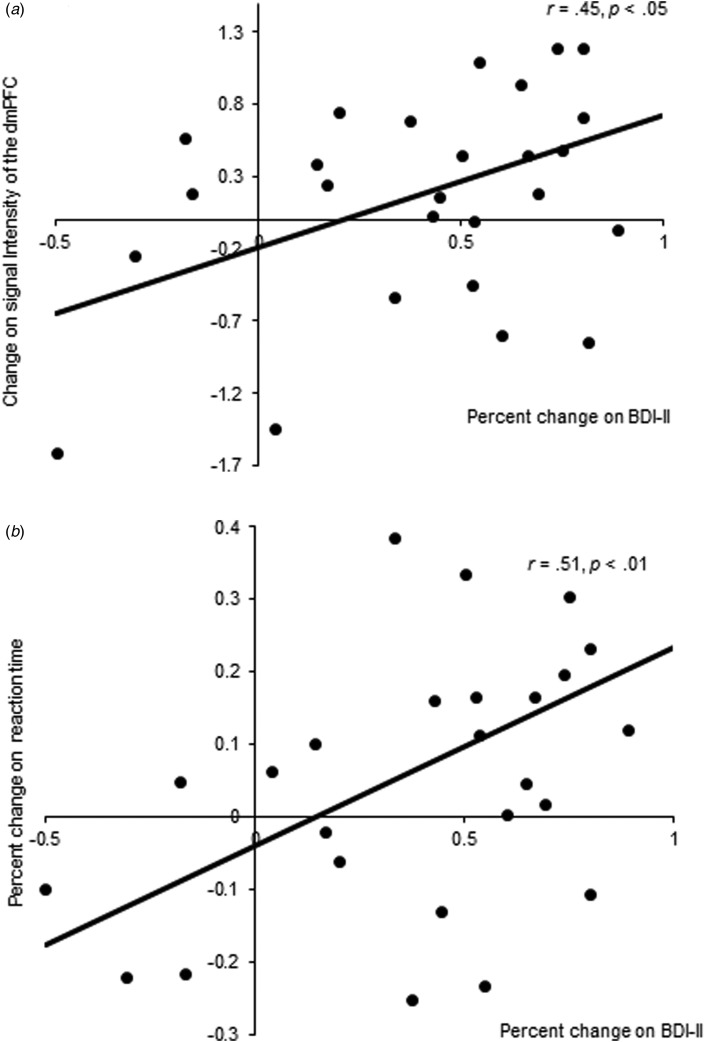
Relationships between variables which show significant intervention effects. Scatter plots and associated correlation coefficients illustrate the relationship between increased dorsal medial prefrontal cortex (dmPFC) activation and percentage change on Beck Depression Inventory-II (BDI-II) scores during other perspective self-judgment condition for positive words (OS-P) in the intervention group (*a*). Scatter plots and associated correlation coefficients illustrate the relationship between percentage change on reaction times and percentage change on BDI-II scores during other perspective self-judgment condition for positive words (OS-P) in the intervention group (*b*).

We extracted the eigenvariate value of the dorsal medial PFC (dmPFC) during OS-P in both the intervention and control groups. The eigenvariate value of dmPFC in OS-P was extracted from subtraction images of contrast using ROI. We conducted a correlation analysis to examine the relationship between Δbrain activation in the dmPFC during OS-P and percentage change on the BDI-II. We found a statistically significant positive correlation between Δbrain activation in the dmPFC and percentage change on the BDI-II in the intervention group (*r* = 0.45, *p* < 0.05, Fig. 2*a*), but not in the control group (*r* = 0.12, *p* = 0.53, n.s.). In addition, we conducted a correlation analysis between percentage change on reaction times and percentage change on BDI-II. We found a statistically significant positive correlation between these indicators in the intervention group (*r* = 0.51, *p* < 0.01, Fig. 2*b*), but not the control group (*r* = 0.15, *p* = 0.45, n.s.).

### Multiple regression

BA increased dmPFC activation and reaction time during OS-P, which was associated with improvements in depressive symptoms. Regions of the mPFC might be related to aspects of cognitive control and scale with reaction time on task (Grinband *et al.* 2008). Therefore, we performed an additional multiple regression analysis during OS-P with Δbrain activation as the dependent variable and percentage change score in reaction times as independent variables, in order to identify activation in cortical regions that were associated with reaction time. As in other analyses, we set the statistical threshold at *p* < 0.001 (uncorrected) at the single voxel level, and *p* < 0.05 (FWE-small volume corrected within the mPFC ROI) at the cluster level. However, there were no areas showing significant positive or negative correlations between reaction time and brain activation during OS-P.

### Functional connectivity change correlated with intervention effect

We performed a generalized pycho-physiological interaction analysis (gPPI; http://www.nitrc.org/projects/gppi: Mclaren *et al*. 2012) with mPFC as the seed region to assess possible functional connectivity related to the intervention effects under the OS condition. Seed regions were defined as radius spheres (6 mm) at specific coordinates based on the results of fMRI group analysis regarding the effect of BA. We created subtraction images of OS-P contrast (post-intervention minus pre-intervention) which were extracted using gPPI of each subject. To determine whether there were any intervention effects on functional connectivity, these subtraction images were compared between intervention and control groups by using a two-sample *t* test. We set the statistical threshold in the whole-brain analysis at *p* < 0.001 (uncorrected) at the single voxel level, and *p* < 0.05 (FWE-small volume corrected) at the cluster level. However, there were no significant differences in connectivity for coupled regions between pre- and post-intervention.

## Discussion

In this study, we found increased activation in the dmPFC in OS-P after BA. Furthermore, there was a positive correlation between this activation increase and improvement of depressive symptoms. Additionally, and only in the OS-P, reaction times for self-referential judgments increased in the intervention group after BA, and there was a positive correlation between improvement of depressive symptoms and this increase. The present study is the first to reveal an effect of BA on other perspective self-reference, at both behaviour and brain-function levels. This pattern suggests that BA enhances adaptive self-monitoring.

Previous studies have suggested that while self-perspective self-reference reflects subjective mental images which arise in a moment (Markus, 1977), other perspective self-evaluation reflects objective mental images which come out through the autobiographical memory retrieval process (Ochsner *et al.* 2005). The self-perspective is assigned primarily during self-referential processing; however, when one evaluates the self from another's perspective, self-perspective self-referential processing is suppressed, and a monitoring function which views the self objectively is promoted (Yokoi *et al.* 2007).

According to Sheppard & Teasdale (2000), healthy individuals show longer reaction times during difficult self-referential judgments, whereas people with depression do not differ in judgment reaction times as a function of difficulty, presumably due to faulty monitoring. Therefore, increased reaction times for other perspective self-referential processing in the intervention group might be related to BA-driven improvement in monitoring function. In BA, planning and executing goal-directed action is a primary focus, such that the treatment might lead to improvement in self-awareness of habitual behaviours or situational triggers, aspects of an objective monitoring function (Lewinsohn & Grosscup, 1980; Jacobson *et al.* 1996).

Recent fMRI studies have suggested that the ventromedial PFC is involved in self-perspective self-referential processing (Yoshimura *et al.* 2010, 2014), and that the dmPFC is involved in other perspective self-referential processing (Ruby & Decety, 2001; Ochsner *et al.* 2005). In particular, our previous research (Yoshimura *et al.* 2014) reported that depressed individuals showed both decreased mPFC activation associated with reduced negative self-reference and increased mPFC activation associated with increased positive self-reference after CBT. We partially replicated these previous findings using BA as an intervention in the present study. In accordance with our prediction, we showed intervention effects on brain activity and behaviour indicators during positive self-reference (OS-P), but contrary to our prediction, not during negative self-reference (OS-N). This pattern might be due to the specific characteristics of the BA intervention as applied in the present study. During BA intervention, individuals might have positive experiences with a corresponding high frequency of environmental rewards and enhancement of monitoring, with improvements in monitoring function likely to be quite specific to such experiences. Therefore, intervention effects might be confined to the OS-P. In the present study, the more depressive symptom improvement a person showed, the longer the OS-P reaction times post-treatment. However, judgment ratio was not associated with depressive symptom improvement. Given these results, we speculated that change in self-concept might occur slowly, through self-evaluation using monitoring function. In contrast, utilization of monitoring function may be established immediately after BA, which might manifest as the increased reaction times we observed. However, as self-concept itself may not have yet changed, judgment ratios remained constant immediately post-treatment.

Regions of the mPFC might be related to aspects of cognitive control and scale with reaction time on task (Grinband *et al.* 2008). To exclude this possibility, we sought to confirm the relationship between inter-individual variability and increased reaction times, and that inter-individual variability increased mPFC activation associated with OS-P. However, there were no significant positive or negative correlations. Therefore, we speculate that increased activation in the mPFC was not related to cognitive control associated with reaction times, and was associated with changes in other perspective self-referential processing.

### Limitations

There are several limitations of this study. First, we did not assess the monitoring function with any self-report questionnaires or observer-rated scales. Although the increased dmPFC activation and judgment latencies suggest improved monitoring function due to BA, quantitative evaluation of monitoring function would be more desirable.

Second, we conducted the present study without using healthy comparison participants. Although we cannot deny the specific effect of intervention, future research should take into consideration these issues. Despite several limitations, to our knowledge, this is the first intervention study to reveal neurobehavioural evidence for the effect of BA on subthreshold depression in self-referential processing.

Third, as shown in the online Supplementary material, there was a significant main effect of group on Δbrain activation in the mPFC. There was significantly increased activation for each contrast in the mPFC of the intervention group than in the control group. We conducted a two-sample *t* test (intervention group *v.* control group) on Δbrain activation in each judgment condition and emotional valence. Moreover, to correct for multiplicity of statistical tests, we report *p* < 0.00625 (0.05/8) for two-sample *t* tests in each condition. Results indicated that the intervention group showed a significant increase in mPFC activation than the control group only in OS-P (*p* = 0.00083). Additionally, we conducted a four-way mixed ANOVA on judgment ratio and reaction times, which indicated a significant interaction in judgment ratio between perspective and valence. Negative valence decreased significantly when using other perspective compared with self-perspective. We also found a significant main effect of reaction time on perspective, as well as a significant interaction between group, time and valence. Reaction time with referential processing using other perspective was longer than referential processing using self-perspective. Furthermore, reaction times increased in the intervention group compared with the control group after BA only in OS-P.

Furthermore, participants in the study were 67% male and 33% female, which was significantly different. According to previous studies, there are no gender differences in the prevalence estimates of subthreshold depression (Kessler & Walters, 1998; Yang *et al.* 2010). In 2014, 2514 freshman were admitted to Hiroshima University, consisting of 1596 males (64%) and 918 females (36%). Of these, we randomly selected freshmen with a BDI-II score greater than or equal to 10, such that the participants were 62.5% male and 37.5% female. Therefore, the gender ratio of the participants of the study reflected the gender ratio of the freshman population in the university in that year.

Finally, we noted the concern about the use of percentage change scores. There are some criticisms about using percentage change scores in parametric tests, because the score often has a non-normal, or a biased distribution. However, using percentage change scores also has the following merits. It can adjust for differences in the range of different variables (Tan & Michel, 2011). Providing information related to the content and precision of scores helps the meaningful interpretation of raw scores (Tong & Kolen, 2010). Analysis of data from clinical trials often uses percentage change scores and raw change scores to adjust treatment responses to the baseline (Kaiser, 1989). For these reasons, we used the percentage change score. The percentage change of BDI-II score and percentage change of reaction time scores in the intervention group and percentage change of reaction time scores in the control group were normally distributed. However, the percentage change score of BDI-II score in the control group was not normally distributed. Therefore, we cannot rule out the possibility that the percentage change score does not typically follow a normal distribution.

## Conclusions

In summary, our results suggest that BA led to increased activation in the dmPFC during other perspective self-referential processing of positive trait words in people with subthreshold depression, which might contribute to improvement in depressive symptoms and objective monitoring function.

## Supplementary material

The supplementary material for this article can be found at https://doi.org/10.1017/S0033291716002956
